# Decrease in the Number of Patients Presenting With ST-Segment Elevation Myocardial Infarction Across Catheterization Centers in Indonesia During the Coronavirus Disease 2019 Pandemic

**DOI:** 10.3389/fcvm.2021.676592

**Published:** 2021-08-16

**Authors:** Doni Firman, Arwin Saleh Mangkuanom, Nanda Iryuza, Ismir Fahri, I Made Junior Rina Artha, Erwin Mulia, Muhammad Syukri, Emir Yonas, Raymond Pranata, Amir Aziz Alkatiri

**Affiliations:** ^1^Department of Cardiology and Vascular Medicine, Faculty of Medicine, Universitas Indonesia, National Cardiovascular Center Harapan Kita, Jakarta, Indonesia; ^2^Department of Cardiology and Vascular Medicine, Mohammad Yunus General Hospital, Bengkulu, Indonesia; ^3^Department of Cardiology and Vascular Medicine, Faculty of Medicine, Udayana University, Sanglah General Hospital, Denpasar, Indonesia; ^4^Department of Cardiology and Vascular Medicine, Bumi Waras Hospital, Lampung, Indonesia; ^5^Department of Cardiology and Vascular Medicine, Faculty of Medicine, Universitas Andalas, DR. M. Djamil General Hospital, Padang, Indonesia; ^6^Faculty of Medicine, Universitas YARSI, Jakarta, Indonesia; ^7^Faculty of Medicine, Universitas Pelita Harapan, Banten, Indonesia

**Keywords:** STEMI, COVID-19, case, decrease, pandemic (COVID-19), cardiovascular

## Abstract

**Background:** The coronavirus disease 2019 (COVID-19) pandemic has become a global problem, put a heavy burden on the health care system, and resulted in many fatalities across the globe. A reduction in the number of cardiac emergencies, especially ST-segment elevation myocardial infarction (STEMI), is observed worldwide. In this study, we aimed to analyze the trends of cases and presentation of STEMI across several cardiac catheterization centers in Indonesia.

**Method:** This retrospective study was performed by combining medical record data from five different hospitals in Indonesia. We compared data from the time period between February to June 2019 with those between February and June 2020. Patients who were diagnosed with STEMI and underwent primary percutaneous coronary intervention (PPCI) procedures were included in the study.

**Results:** There were 41,396 emergency department visits in 2019 compared with 29,542 in 2020. The number of patients with STEMI declined significantly from 338 in 2019 to 190 in 2020. Moreover, the total number of PPCI procedures reduced from 217 in 2019 to 110 in 2020. The proportion of PPCI was not significantly reduced (64.2 vs. 57.9%). The majority of the patients were men, with a mean age of 54 years in 2019 and 55 years in 2020. We observed a significantly longer door-to-balloon time in 2020 than in 2019 (*p* < 0.001). We also observed a difference in the door-to-balloon time and ischemic time between the two periods.

**Conclusion:** We observed a decline in the number of patients presenting with STEMI to our centers. However, we observed no significant decline in the percentage of PPCI performed across our centers during this pandemic.

## Introduction

The coronavirus disease 2019 (COVID-19) pandemic has become a global problem that has put a heavy burden on the health care system and resulted in many fatalities (1, 2). Each country has different policies to limit the transmission of COVID-19. When the disease began to spread in Indonesia in February 2020, many health services were disrupted because hospitals or other health care facilities had to change their service system policies to reduce the spread of the virus.

The pandemic also resulted in a significant reduction in the number of patients seeking medical care either in the emergency department or outpatient clinic. A reduction in the number of cardiac emergencies, especially ST-segment elevation myocardial infarction (STEMI), was observed worldwide (3–9). Data on 115,716 adult emergency department visits to 108 emergency departments in the United States showed a significant reduction in the incidence of most of the serious cardiovascular events, with the exception of STEMI, in the year 2020 compared with that in 2019 (10). Similarly, no decrease in the number of patients presenting with STEMI was observed in France, and the authors of the study hypothesized that the pandemic probably dissuaded “non-critical” patients, but not those requiring reperfusion (11). In Greece, the number of patients presenting to the emergency department with acute coronary syndrome (ACS) in 2020 was significantly reduced compared to that in the previous year (12). Similarly, the number of patients presenting with cardiovascular emergencies in Germany decreased in 2020 (13). The number of immediate/early percutaneous coronary interventions (PCIs) for non-ST-segment elevation myocardial infarction (NSTEMI) in China significantly decreased in 2020 compared to that in the previous year (14).

Several possible causes for a decrease in the number of STEMI cases in Indonesia have been proposed; one of them is the fear of visiting the hospital during the COVID-19 pandemic. In this study, we aimed to analyze the trends of cases and presentation of STEMI across several cardiac catheterization centers in Indonesia.

## Materials and Methods

This was a retrospective study involving 190 patients with STEMI in 2020 and 338 patients in 2019. This study was approved by the Ethical Committee of the National Cardiovascular Center Harapan Kita (LB 02.01/VII/KEP 070/2018) and adhered to the declaration of Helsinki. Data were obtained from five hospitals in Indonesia, namely the National Cardiovascular Center Harapan Kita (Jakarta, Indonesia), Mohammad Yunus General Hospital (Bengkulu, Indonesia), DR. M. Djamil General Hospital (Padang, Indonesia), Bumi Waras Hospital (Bandar Lampung, Indonesia), and Sanglah General Hospital (Denpasar, Indonesia). These hospitals are referral centers for PCI and cardiovascular care in their respective regions. The National Cardiovascular Center Harapan Kita is a national tertiary hospital for cardiovascular disease, while the rest are general hospitals with cardiac intervention facilities. We retrieved medical records data from two different periods: (i) between February 1, 2019, and June 30, 2019; and (ii) between February 1, 2020, and June 30, 2020. Patients who were diagnosed with STEMI and underwent primary PCI (PPCI) procedures were included in the study. We also included data of patients with STEMI without PPCI and overall presentation to the emergency departments of the participating hospitals for comparative purposes.

During the second observation period of this study, most of the patients underwent rapid test for COVID-19 antibody. The number of polymerase chain reaction tests performed on these patients was very small due to the unavailability at the time. Thus, the diagnosis of COVID-19 was mainly based on rapid test for COVID-19 antibody. The data retrieved included demography, medication, procedural data, in-hospital outcome, length of hospital stay, angiographic characteristics, total ischemic time, patient's delay, hospital, and system delay. Thrombolysis in myocardial infarction (TIMI) flow grade was assessed angiographically by the physician performing the procedure. During the PCI procedure, door-to-wire crossing time was recorded as a surrogate for reperfusion per the 2017 European Society of Cardiology guidelines for the management of acute myocardial infarction (AMI) in patients presenting with ST-segment elevation. Continuous variables are presented as median [25th percentile, 75th percentile]. Categorical variables are presented as frequency and percentage.

In this paper, we present continuous data as median, minimum and maximum, and mean + standard deviation as appropriate. Percentage is used to present discrete data. We used Statistical Package for the Social Sciences (SPSS) software version 25 for Windows (SPSS, Chicago, Illinois, USA). Baseline analysis of patients' demography and comorbidity. The independent samples *t*-test, the chi-square test, and Fisher's exact test were used to compare variables as appropriate.

We compared sex, age, diabetes, hypertension, smoking status, and Killip class between 2019 and 2020 data. Clinical parameters and procedural data were analyzed using the *t*-test to compare percentages, onset, time interval since STEMI onset, door-to-balloon time, ischemic time, and LVEF. We also analyzed adverse effects and complications including bleeding, initiation of CPR, stroke, mortality, and length of hospital stay.

## Results

The baseline characteristics are illustrated in Tables 1, 2, the complications and outcomes are presented in Table 3. There were 41,396 and 29,542 emergency department visits in 2019 and 2020, respectively. The number of patients with STEMI declined significantly from 338 cases in 2019 to 190 cases in 2020. Moreover, the number of PPCI procedures decreased from a total of 217 in 2019, to 110 in 2020. The majority of the patients were men, with a mean age of 54 years in 2019 and 55 years in 2020 as shown in Table 1. There were no significant differences regarding the presence of hypertension and diabetes mellitus between the two periods. In the pandemic period, the number of patients who presented directly to the PCI center's emergency department declined sharply compared to referred patients from the other health care facilities (*p* < 0.001). Furthermore, the onset of symptoms to arrival was found to be similar in between the two periods. Nevertheless, we observed a significantly longer door-to-balloon period and ischemic time in the pandemic period than in 2019 (*p* < 0.001 and *p* = 0.041, respectively) (Tables 1, 2).

**Table 1:** Baseline characteristics of all study subjects.

**Characteristic**	**2019 (*n* = 338)**	**2020 (*n* = 190)**	***P*-Value**
Male (%)	294 (87)	167 (87.9)	0.787
Age, mean (SD), years	55.31 (11.34)	55.17 (10.50)	0.888
Hypertension (%)	196 (58.0)	103 (54.20)	0.412
Diabetes (%)	131 (38.8)	79 (41.6)	0.578
Smoker (%)	193 (57.1)	85 (44.7)	0.007
Killip Class I (%)	197 (58.3)	103 (54.2)	0.092
**Presentation of Cases (Overall)**			
Patient self-presenting to PCI Capable Center (%)	178 (52.7)	64 (33.7)	<0.001
Referred from other hospital (%)	160 (47.3)	126 (66.3)	<0.001
**Procedures for patients with STEMI**			
Conservative treatment (%)	55 (16.3)	33 (17.4)	0.299
Fibrinolysis (%)	66 (19.5)	47 (24.7)	0.299
Primary PCI (%)	217 (64.2)	110 (57.9)	0.299

**Table 2:** Baseline and procedural characteristics of study subjects who underwent primary PCI.

**Characteristic**	**2019 (*n* = 217)**	**2020 (*n* = 110)**	***P-*Value**	**Difference between means (95% CI)**
Male (%)	194 (89.4)	97 (88.2)	0.713	
Age, mean (SD), years	54.01 (10.19)	55.00 (10.06)	0.405	
Hypertension (%)	137 (63.1)	73 (66.3)	0.326	
Diabetes (%)	125 (57.6)	71 (64.5)	0.235	
Smoker (%)	140 (64.5)	51 (26.8)	<0.001	
Killip Class I (%)	197 (90.8)	103 (93.6)	0.257	
**Presentation of Cases**				
Patient self-presenting to PCI Capable center (%)	126 (58.1)	38 (34.5)	<0.001	
Referred from other hospital (%)	91 (41.9)	72 (65.5)	<0.001	
**Clinical Parameters**				
STEMI Onset, mean (SD), minutes	349.60 (158.48)	363 (171.24)	0.611	13.4 (−50.9–24.1)
Door-to-balloon time, mean (SD), minutes	97.79 (60.29)	125.56 (66.35)	<0.001	27.7(−42.1–13.4)
Ischemic Time, mean (SD), minutes	447.33 (164.75)	488.57 (185.26)	0.041	41.24 (−80.81–1.65)
LVEF, Mean (SD)	48.13 (12.86)	50.69 (13.59)	0.199	0.2 (−5.7–0.6)
Anterior MI (%)	116 (53.5)	56 (50.9)	0.317	
**Procedural Data**				
Radial (%)	171 (78.8)	92 (83.6)	0.243	
TIMI 0 Flow, Pre (%)	181 (83.4)	93 (84.5)	0.638	
TIMI 3 Flow, Post (%)	180 (82.9)	103 (93.6)	0.027	
Stent number, mean (SD)	1.14 (0.38)	1.15 (0.41)	0.844	

**Table 3:** Outcomes of interests.

**Complication and Outcome**	**2019 (*n* = 338)**	**2020 (*n* = 190)**	***P-*Value**	**Difference between means (95% CI)**
Bleeding, Yes (%)	8 (2.4)	2 (1.1)	0.505	
CPR, Yes (%)	14 (4.1)	9 (4.7)	0.648	
Stroke Event, Yes (%)		1 (0)	0.336	
Mortality, Alive (%)	206 (60.9)	104 (54.7)	1.000	
Length of Stay, mean (SD), days	5.27 (3.12)	5.29 (3.15)	0.935	0.02 (−0.74–0.69)

**Figure 1:**
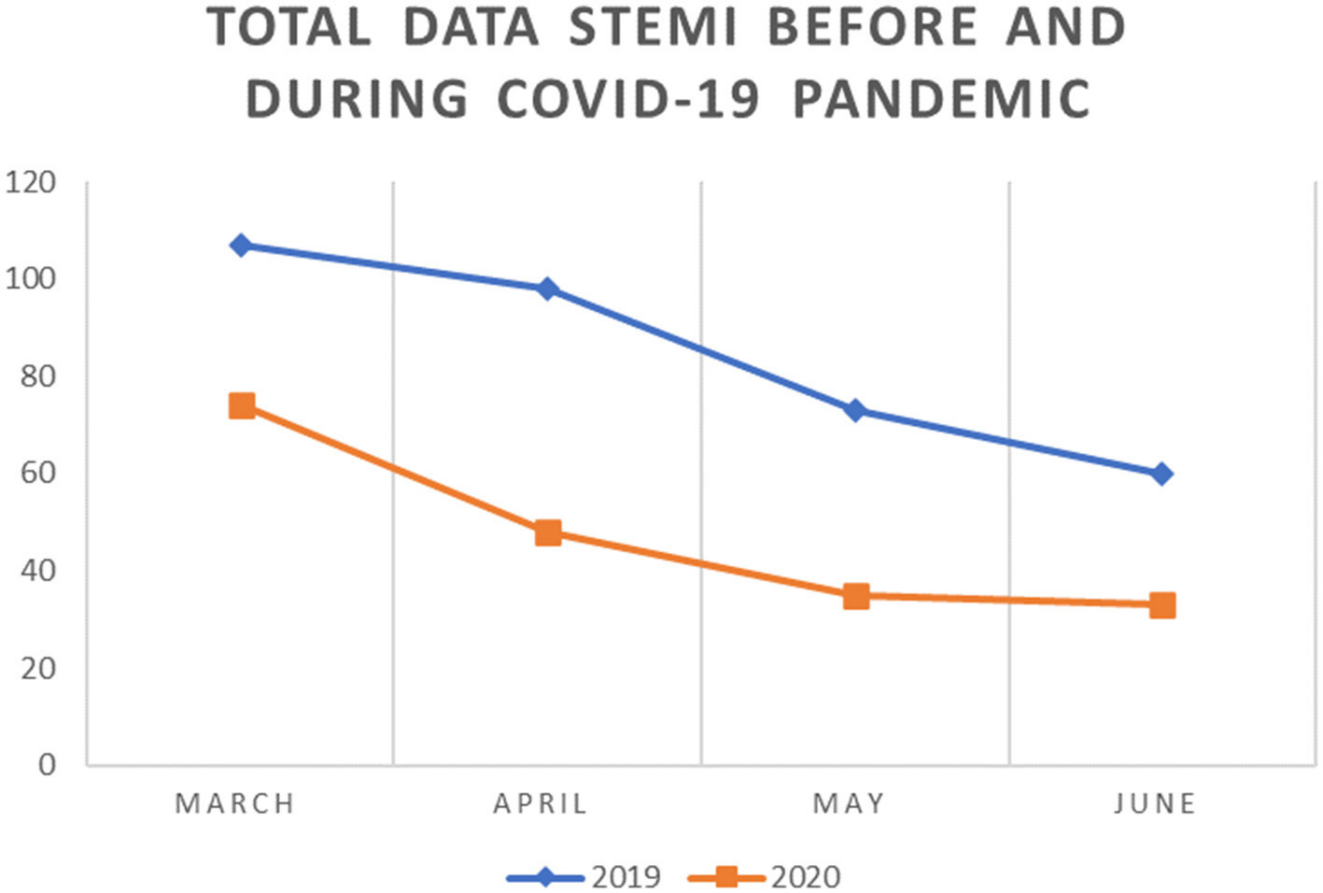
Total STEMI Cases before and during COVID 19 pandemic.

**Figure 2:**
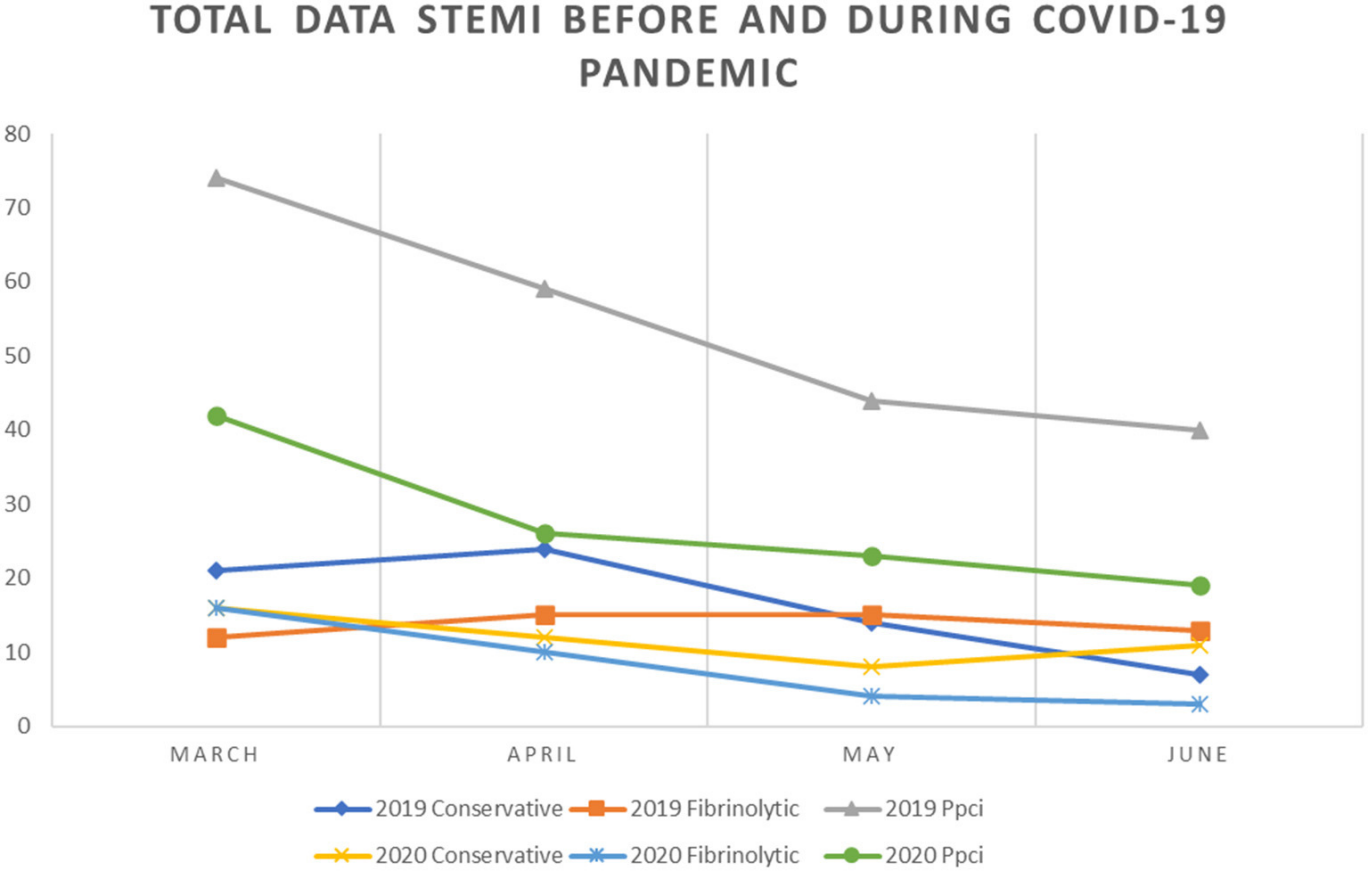
Comparison between conservative, fibrinolytic and primary PCI management of STEMI in 2019 and 2020.

**Figure 3:**
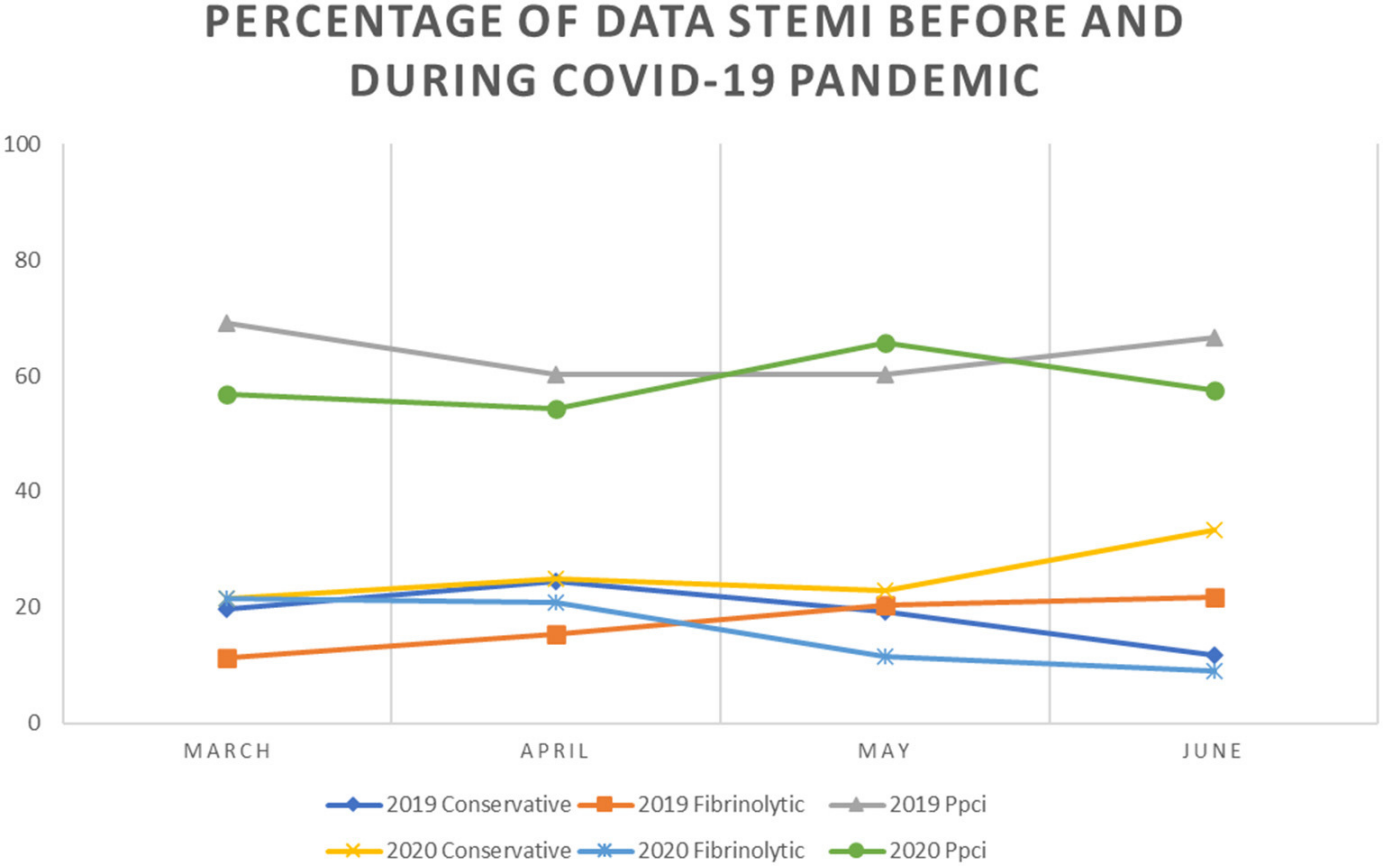
Percentage of STEMI Cases before and during COVID 19 Pandemic.

We observed a trend of decrease in the number and percentage of STEMI cases and rates of PPCI in 2020 compared with 2019. A total of 74 cases with STEMI were reported from the five catheterization centers in March 2020 compared to 107 cases in March 2019 and the trend continues until the end of observation in June. Overall, compared with the number of patients with STEMI in 2019, we noted a trend for decrease in 2020, reaching the lowest point in June 2020 when there were only 34 patients with STEMI compared to 60 cases in June 2019. We observed no significant change in the percentage of PPCI performed across our centers. However, the percentage of patients undergoing conservative treatment was higher in June 2020 compared with that in the previous year (33.3 and 11.7%, respectively) (Figures 1–3).

## Discussion

During the COVID-19 pandemic, a significant decrease in the number of hospitalizations of patients with ACS was seen in the United Kingdom (UK), Ireland, Italy, and the United States. Data from NHS facilities across the UK show a hospitalization rate reduction of 40% for ACS, 35% for AMI, 23% for STEMI, and 42% for NSTEMI. Similar observations were reported from Italy, with ~50% reduction in patients with AMI presenting to the hospital in every region of the country. Moreover, a 26.5 and 65.1% reduction in patients with STEMI, and NSTEMI, respectively, and a significantly increased case fatality rate for STEMI compared with those in the previous year were observed (RR 3.3, 95% confidence interval [CI] 1.7–6.6; *P* < 0.001) (8, 15–18).

A lower number of patients with STEMI presented at our PCI centers during the pandemic. Therefore, we might assume that patients were reluctant to come to a hospital with a PCI center, which usually is larger and more crowded with patients, to avoid COVID-19 transmission and tended to visit a less crowded hospital or other health care providers. Compared with 2019, we observed an increase in the door-to-balloon time (97.79 vs. 125.56 min, *p* < 0.001) and ischemic time (447.33 vs. 488.57 min, *p* = 0.041) in 2020. Lockdown and stay at home order may cause patient delay in seeking medical care, Indonesia did not impose lockdown, which may explain only small difference (although statistically significant) between the ischemic time in 2019 and 2020. Solomon et al., in a study using data from the Kaiser Permanente Oakland Medical Center, observed a decrease in the number of hospitalizations of patients with STEMI in conjunction with the first reported death due to COVID-19 in California, 2 weeks before the implementation of the shelter in place order (18). This finding shows that social restriction policies might play a smaller role than previously believed in decreasing the rate of presentation to hospitals. Contrary to the observations in the US, in the data from NHS facilities across the UK, a decrease in admissions for ACS occurred at least 2 weeks before the first death due to COVID-19. In response to this, the British Heart Foundation and the British Cardiovascular Society launched public campaigns to encourage patients with heart attack symptoms to visit a hospital (17).

We observed a reduction in the proportion of smokers among admitted patients with STEMI in 2020 compared with that in 2019 (57.1 vs. 44.7% *p* = 0.007). In our view, this does not necessarily signify a reduction in the number of smokers, but rather it is an index of the reduction in the number of patients admitted to PCI centers. As supported by the finding of a reduced percentage of patients who presented themselves to the hospital for STEMI in 2020 compared with that in 2020 (34.5 vs. 58.1%, *p* < 0.001), the majority of admitted patients were referred from other hospitals during the COVID-19 pandemic (41.9 vs. 65.5%, *p* < 0.001), which showed an increase from that in 2019. In contrast, during 2019, the majority of admissions for PPCI were voluntary presentations to PCI centers. Our findings were similar to those of a study by Mafham et al. regarding the decrease in admissions for STEMI in the UK during the COVID-19 pandemic. Our findings show the reluctance of patients with STEMI to voluntarily seek medical care and present themselves to cardiovascular centers compared with the previous year, possibly due to fear of contracting COVID-19. Mafham et al. also associated this reduction in patients with STEMI in their study with the increased news coverage of COVID-19 in the media, and supported the hypothesis that this is caused by the widespread fear of COVID-19 (17).

We found markedly longer door-to-balloon time in 2020 compared with that in 2019 (125.56 vs. 97.79 min, *p* < 0.001). This delay was due to several reasons. First, patients were examined thoroughly for signs and symptoms of COVID-19 before entering the ED. Second, after patients entered the ED, several examinations were performed to minimize the risk of COVID-19 transmission such as completing the COVID-19 epidemiological form, chest x-ray scan, complete blood count, rapid immunological test for COVID-19, and chest CT-scan if necessary. Third, since most hospitals in Indonesia were not well-prepared for a dedicated isolated catheterization laboratory (cath lab), the donning process of the personal protective equipment (PPE) for the operator and scrub nurses might have prolonged the door-to-balloon time. Furthermore, we acknowledge that the exercise of precautions, such as travel and contact history interviews and chest x-ray images taken before patient transfer to the cath lab, may be possible causes of delay. However, this measure is crucial, especially because most cath labs are positively ventilated and performing procedures in this environment might facilitate the spread of disease if patients are not screened thoroughly (5).

Our observation regarding the increase in door-to-balloon time during the COVID-19 pandemic was consistent with results of a study by Tam et al., who also observed an increase in the symptom to the first medical contact time, door to device time, and cath lab arrival to device time compared with those in the years 2018 and 2019. The authors reported a minimum increase of 226.5 min from symptom onset to first medical contact time, and a minimum of increase of 11 min in the cath lab arrival to device time. Interestingly, in non-office-hours presentations, a decrease in the door to device time compared to that in previous years was noted.

In these trying times, we observed a shift in reperfusion strategies for treating STEMI while adapting to the highly contagious nature of COVID-19. A protocol from Sichuan provincial hospital advocates thrombolysis in an isolation ward for all COVID-19 positive patients with STEMI, with no contraindication for thrombolysis in patients with onset times within 12 h; an elective PCI is considered subsequently for this group of patients. Stable patients with onset time >12 h are evaluated for PCI, while unstable patients with severe pneumonia are given conservative treatment in an isolation ward (19). Similar guidelines, primarily focusing on the utilization of thrombolysis for COVID-19 positive patients with STEMI, were also proposed by the Peking Union Medical College Hospital, while for NSTEMI patients, they recommended that the treatment of COVID-19 pneumonia by an infectious diseases specialist should take precedence (20). However, guidelines from the Society for Cardiovascular Angiography and Interventions (SCAI) Emerging Leader Mentorship advocate PCI for COVID-19 positive patients with STEMI, albeit with several considerations such as screening before arrival at the cath lab and optimal use of PPE for staff. In its guidelines, SCAI acknowledges the possibility of overload of the healthcare system during this pandemic; however, systemic fibrinolytic therapy is only advocated for low-risk STEMI (inferior STEMI without right ventricular involvement or lateral myocardial infarction without hemodynamic compromise). For patients with high-risk STEMI, SCAI advocates the use of PCI as the primary modality; however, it should only be performed on the culprit vessel. PCI should be performed on a non-culprit vessel only if the lesion is deemed unstable or in the case of multiple culprit vessels (21). The recommendation of SCAI is in accordance with that of The European Society of Cardiology in their European Association of Percutaneous Coronary Intervention (EAPCI) position statement on invasive management of ACS during the COVID-19 pandemic. In this statement, PPCI remains the preferred reperfusion strategy in PCI centers, provided that it fits within the time frame (120 min from onset of symptoms). EAPCI also states that all patients with STEMI should be managed as COVID-19 positive. If considerable delay in reperfusion strategy is anticipated due to implementation of protective measures, fibrinolysis should be performed, given that there are no contraindications. EAPCI also recommends complete revascularization when appropriate and left ventricular angiogram in place of echo to evaluate left ventricular function (22). Additionally, a position statement from SCAI, ACC, and ACEP also recommend PPCI as the standard of care in patients with STEMI (23). We observed a statistically significant slight increase in the proportion of patients with post procedural TIMI3 flow (53.3 vs. 54.2%, *p* = 0.027). Total ischemic time and time-to-treatment delays for urgent cardiovascular interventions are expected to be longer during the pandemic as a consequence of screening and other policies related to the pandemic (24). This finding was also noted in the present study, wherein there was an increase in ischemic time during the period of study in 2020 compared with that during the period of study in 2019 (488.57 vs. 447.33 min, *p* = 0.041).

Currently, at all catheterization centers in our study, we perform PPCI on all COVID-19 positive patients with STEMI with onset <12 h. We also perform PPCI on patients with STEMI with onset >12 h who are clinically unstable. Currently, catheterization of all patients with ACS is performed in dedicated specialized isolated cath labs. After PCI, patients with reactive severe acute respiratory syndrome coronavirus 2 antibody tests, absolute lymphopenia, neutrophil to lymphocyte ratio >3.13, C-reactive protein >10, or infiltration on chest x-ray scanning are admitted to the isolation ward, whereas patients with none of the above are admitted to the regular ward.

Although we observed trends for lower rates of bleeding and C-reactive protein in this study, we did not observe an increase in mortality during the COVID-19 pandemic compared with that in the previous year (5.5 and 5.1% for 2020 and 2019, respectively, *p* < 0.001). In contrast, a study from Italy observed a significant increase in fatality rates of STEMI during the COVID-19 pandemic (13.7 and 4.1% for 2020 and 2019, respectively, RR = 3.3, 95% CI: 1.7–6.6; *P* < 0.001) (15).

The reduction in the presentation and admission rates for STEMI in Indonesia and other countries might not represent a true decrease in incidence of STEMI in the general population. This phenomenon might represent reluctance of patients to seek medical care due to fear of contracting COVID-19. We observed that self-presenting patients decreased while transfer patients increased, both for total number and the percent with PCI, this be due to the characteristics of the hospitals. Hospitals included in this study are large government referral hospital and were assigned as COVID-19 treatment hospitals. It is possible that patients were more reluctant to present to these hospitals and chose less crowded hospitals that turns out to be non-PCI capable hospital, thus reducing self-presentation to our designated hospitals while concomitantly increasing the number of referrals.

Public health counseling of the community by medical practitioners plays an important role in combating the paranoia of contracting COVID-19 in case of an emergency. Patients with complaints suggestive of underlying myocardial infarction will gain the most benefit from timely medical attention to prevent sequelae in the future (25). Patients with STEMI will gain the most benefit of timely medical attention, since this subgroup of patients has the highest risk of out of hospital cardiac arrest without proper medical treatment (6, 26, 27).

Currently, Indonesia imposes no lockdown on its population. However, large scale social restrictions were imposed nationally and regionally by provincial governments in areas with a high prevalence of COVID-19. They included measures such as closing public places, restricting public transport, and limiting travel to and from the restricted regions. This policy was enacted in March 2020 after confirmation of 117 cases of COVID-19 on the 15th of March 2020; the first case of COVID-19 in Indonesia was reported on the 2nd of March 2020.

The enactment of this policy, which restricts travel, might have hindered patients from reaching cardiovascular centers for proper management of their complaints. Reports from the UK also indicate that the enforcement of lockdown/social distancing measures might further influence public perception into the reluctance of going to medical centers even when the need arises. This phenomenon might be what is currently happening in Indonesia. Ideally, efforts should be made to encourage the public to seek proper medical care when the need arises (17). Another potential cause of decreased STEMI and other acute cardiovascular diseases is the reduced pollution and change in lifestyle due to “work from home” and other policies (28, 29).

### Study Limitation

Due to the abrupt and fast increase in COVID-19 cases in Indonesia during the study period (February–June 2020), our centers were not prepared for COVID-19 testing and diagnosis at the outset of this pandemic. Testing kits using Reverse Transcriptase Polymerase Chain Reaction were unavailable until August 2020, while rapid testing for COVID-19 using serum immunoglobulin assay was available only at certain centers. In most cases, an epidemiological contact form was used to supplement these testing kits. Unfortunately, we were unable to obtain precise data regarding the results of rapid tests.

In conclusion, we observed a decline in the number of patients presenting with STEMI to our centers. However, we observed no significant decline in the percentage of PPCI being performed across our centers during this pandemic. We also observed prolongation of the door-to-balloon time and ischemic time compared with those in the same month in the previous year. Although delays might be harmful to patients, this delay was caused by preliminary screening of patients with STEMI for COVID-19 at the emergency department.

## Data Availability Statement

The raw data supporting the conclusions of this article will be made available by the authors, without undue reservation.

## Ethics Statement

The studies involving human participants were reviewed and approved by Ethical Committee of the National Cardiovascular Center Harapan Kita (LB 02.01/VII/KEP 070/2018). The ethics committee waived the requirement of written informed consent for participation.

## Author Contributions

DF: conceptualization, methodology, and writing—review and editing. AM, IF, IA, EM, and MS: writing—review and editing. NI: formal analysis. EY and RP: writing—original draft, writing—review and editing, and visualization. AA: supervision, project administration, and writing—review and editing. All authors contributed to the article and approved the submitted version.

## Conflict of Interest

The authors declare that the research was conducted in the absence of any commercial or financial relationships that could be construed as a potential conflict of interest.

## Publisher's Note

All claims expressed in this article are solely those of the authors and do not necessarily represent those of their affiliated organizations, or those of the publisher, the editors and the reviewers. Any product that may be evaluated in this article, or claim that may be made by its manufacturer, is not guaranteed or endorsed by the publisher.
